# Exploring the impact of vitamin D-related genetic variants on muscular fitness changes in middle-aged and older adults in Kosovo

**DOI:** 10.3389/fpubh.2025.1476492

**Published:** 2025-02-13

**Authors:** Ermira Krasniqi, Arben Boshnjaku, Karl-Heinz Wagner, Barbara Wessner

**Affiliations:** ^1^Research Platform Active Ageing, University of Vienna, Vienna, Austria; ^2^Vienna Doctoral School of Pharmaceutical, Nutritional and Sport Sciences (PhaNuSpo), University of Vienna, Vienna, Austria; ^3^Department of Sport and Human Movement Science, Centre for Sport Science and University Sports, University of Vienna, Vienna, Austria; ^4^Faculty of Medicine, University “Fehmi Agani” in Gjakova, Gjakovë, Albania; ^5^Department of Nutritional Sciences, Faculty of Life Sciences, University of Vienna, Vienna, Austria

**Keywords:** physical performance, 25-hydroxyvitamin D, GC, VDR, polymorphism, aging

## Abstract

**Introduction:**

Age-related decline in muscle strength and performance significantly impact morbidity and mortality. Various factors including genetics have been investigated to better understand this decline. This study aimed to investigate longitudinal changes in physical performance and strength and their association with genetic variants in genes involved in the vitamin D pathway.

**Methods:**

This longitudinal study was conducted in the Prishtina region, Kosovo, with community-dwelling adults over 40 years of age. Genomic DNA was extracted from saliva samples to assess single nucleotide polymorphisms in the vitamin D receptor (VDR) gene (rs7975232, rs2228570, rs731236, also referred to as ApaI, FokI, and TaqI, respectively) and the vitamin D binding protein (GC) gene (rs4588, rs2282679). Physical performance was assessed by isometric handgrip strength, 30-s chair stand, timed up and go and 6-min walk test. Vitamin D levels were assessed from blood samples only at follow-up.

**Results:**

A total of 138 participants (65.1 ± 9.0 years, 52.2% female) were included. Over a 2.7-year period, significant declines in the 30-s chair stand test (*p* < 0.001) and timed up and go performance (*p* < 0.001) were observed, whereas BMI increased. Only female participants experienced a decrease in handgrip strength (*p* < 0.001). Genotyping showed significant associations of the ApaI variant with changes in BMI and handgrip strength. Participants with the minor CC genotype showed a greater increase in BMI and a greater decrease in absolute and relative handgrip strength. No significant interactions were observed for FokI and TaqI in the VDR gene, or rs4588 and rs2282679 in the GC gene. Vitamin D deficiency (<50 nmol/L) was prevalent in 47.5% of participants, with significant differences in 25(OH)D levels observed between genotypes of the GC gene (rs4588, *p* = 0.039; rs2282679, *p* = 0.036).

**Conclusion:**

Physical fitness declined significantly over time, with female participants experiencing a greater decline in handgrip strength. The ApaI variant in the VDR gene was associated with changes in muscle strength, while variants in the GC gene were associated with vitamin D levels. These findings suggest that genetic factors related to the vitamin D pathway may contribute to the age-related decline in muscle strength. Therefore, genetic predisposition should be considered when developing individual interventions for healthy aging.

## 1 Introduction

The aging process is characterized by numerous physiological changes throughout an individual’s life. A noteworthy aspect of this process is the decline in muscle strength, which is reported to commence as early as the third or fourth decade of life (1). Muscle strength and physical performance are often considered the most reliable indicators of age-related muscle alterations (2). The revised consensus criteria for defining and diagnosing sarcopenia by the European Working Group in Sarcopenia for Older People (EWGSOP2) have suggested to focus on low muscle strength as a key feature of sarcopenia, followed by low muscle quantity and quality for confirming the sarcopenia diagnosis, and poor physical performance for establishing the severity of sarcopenia (3).

Numerous factors and patterns are being investigated for their potential association with the aging process, particularly about muscle strength and function (4). Genetics stands out as an area of interest in exploring its potential influence on age-related muscle strength and function. One specific focus has been on the impact of gene polymorphisms related to the vitamin D pathway, their potential effects on vitamin D status, as well as muscular characteristics (5).

Some of these studies have focused specifically on the VDR gene, which encodes the vitamin D receptor and whose muscular expression has been shown to decline with age (6). In the VDR gene, the rs2228570 (FokI) polymorphism produces a protein that is three amino acids shorter, while rs731236 (TaqI) and rs7975232 (ApaI) are in a high linkage disequilibrium with 3` UTR polymorphisms (7). These polymorphisms are not involved in protein coding but may influence mRNA stability due to their proximity to the poly (A) tail (8). Another interesting gene within the vitamin D/muscle axis is the group-specific component (GC) gene encoding the vitamin D binding protein, which binds nearly all 25-hydroxyvitamin D (25(OH)D) in the blood and has a high affinity for actin in skeletal muscle cells (9). Carriers of the variant A allele of the commonly studied rs4588 polymorphism in the vitamin D binding protein not only show a lower affinity of the vitamin D binding protein to 25(OH)D, but also lower levels of both, vitamin D binding protein and 25(OH)D (10). Another variation within the GC gene, rs2282679, which is in strong linkage disequilibrium with rs4588, is associated with vitamin D status (5).

In a first cross-sectional study conducted in community-dwelling adults from the Prishtina region (Kosovo), we showed that the prevalence of probable sarcopenia, sarcopenia and severe sarcopenia was dependent on the specific cut points used, but was 5.5, 5.5, and 2.4% in men, respectively, according to the EWGSOP2 criteria. Possibly masked by the high prevalence of overweight and obesity, no cases of sarcopenia and severe sarcopenia were detected in women (11). A common ACTN3 polymorphism (rs1815739), frequently associated with muscle phenotypes, was not convincingly associated with muscle mass, strength and performance (12, 13). Three years later, we performed a second study focusing on vitamin D status. Vitamin D deficiency [25(OH)D concentration < 50 nmol/L] was observed in 47.5% of middle-aged and older adults, and low serum 25(OH)D was associated with low muscle strength (14). As a sub-sample of the first study participants also enrolled in the second study, this secondary analysis aimed to investigate whether longitudinal changes in muscle characteristics would be related to the frequently reported polymorphisms across the VDR gene (rs7975232 (ApaI), rs2228570 (FokI) and rs731236 (TaqI)) and the GC gene (rs4588 and rs2282679). By examining the relationship between these genetic variants and longitudinal changes in muscle characteristics, the study hopes to contribute to our understanding of how genetic predisposition may affect muscle health in older adults. We hypothesized that unfavorable variants in the VDR or GC genes would be associated with a greater decline in muscle mass, strength, and performance.

## 2 Methods

### 2.1 Study population

**Figure 1:**
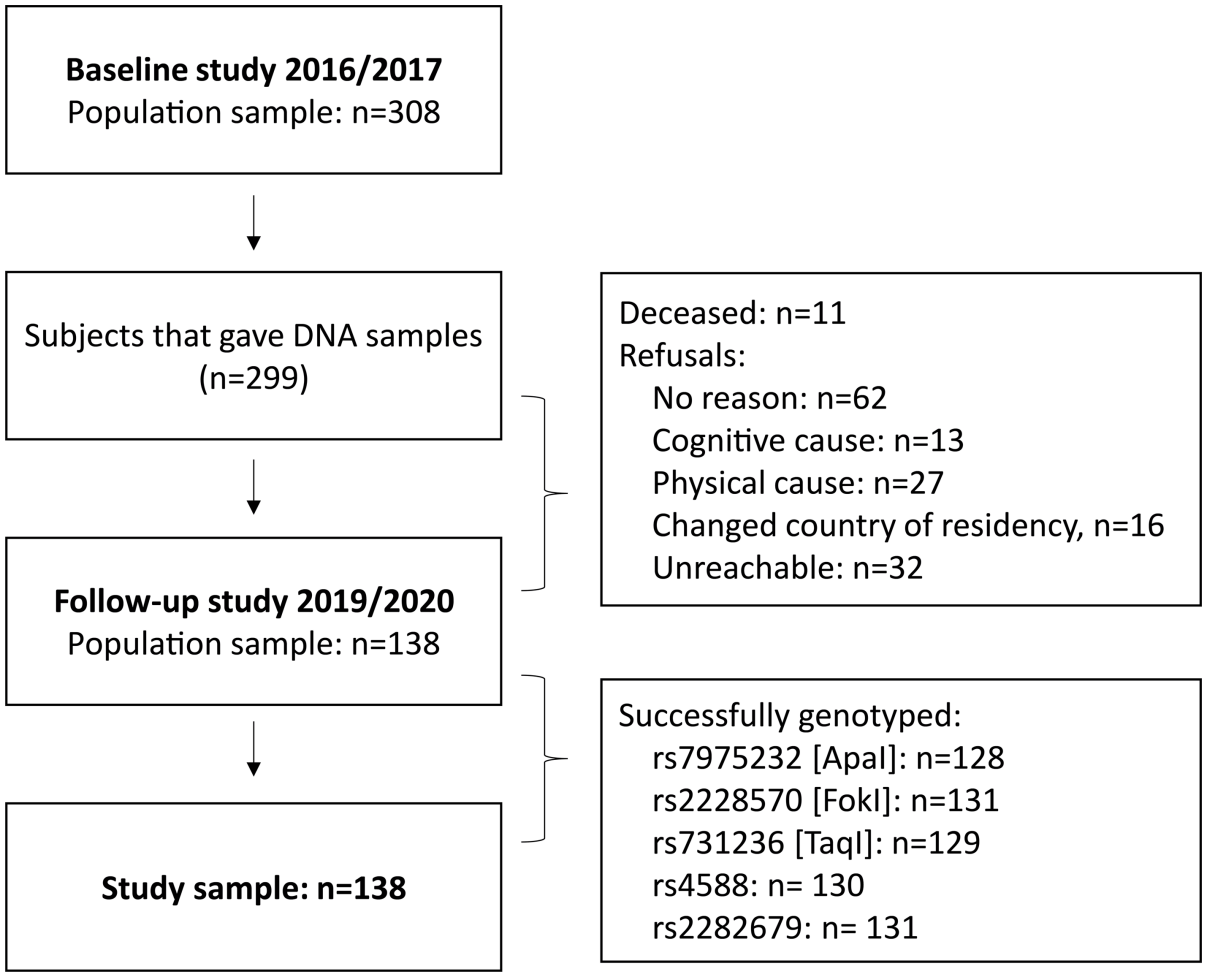
Participant flow.

This longitudinal observation used data from two cross-sectional studies conducted in two different time periods: August 2016 – June 2017 and December 2019 – February 2020 (Figure 1). From the 308 subjects participating in the first study, 138 agreed and were eligible to participate in the second follow-up study. The recruitment strategy and inclusion criteria for the initial studies were described in detail in a previous publication (12). The participants were initially selected through purposive sampling, with the aim of ensuring that they met the specific criteria that were relevant to the study objectives. Additionally, we used snowball sampling, where existing participants referred others from their networks who met the study criteria. This approach allowed us to leverage the close-knit relationships common in the area, which helped us to expand our participant pool and enhance the relevance of the insights gathered. The criteria ensured that male and female participants were over 40 years of age, with no upper limit, had no acute illnesses or conditions that would directly affect their ability to participate in the physical performance tests, and belonged to the specified region and age group. The distribution of male and female participants was based on the natural availability and eligibility of volunteers within the target demographic, rather than a strict quota. By prioritizing eligibility criteria, we aimed to ensure that our sample accurately reflected the demographic characteristics of the population, thereby increasing the validity of our findings.

The first measurements took place at the Sports Medicine Laboratory of the Universi College in Prishtina (Kosovo), while the second measurements took place at the Laboratory for Human Biomarkers of the University “Fehmi Agani” in Gjakova (Kosovo). Both studies were conducted by the same research group, strictly following the initially set order (personal health, behaviors and socio-economic data collection, anthropometric measurements, body composition, short 30–60 min lunch break with a standardized light meal and physical performance assessments). At the second assessment, blood samples were taken by licensed health professionals in the morning immediately after the personal data collection.

Each participant followed a specified protocol, which began the day before with abstinence from alcohol and overnight fasting, as well as wearing light indoor clothing during the assessments, in accordance with the International Standards for Anthropometric Assessments (15). Measurements started with personal data collection, saliva (study 1) or blood (study 2) sampling, anthropometry, body composition, a short break with a light standardized meal, and concluded with physical performance assessments. Nutritional status score was assessed using the long form of the Mini Nutritional Assessment (MNA) questionnaire (16).

### 2.2 Anthropometric and physical fitness assessments

Anthropometric and physical fitness assessments have been described previously (11, 14). Briefly, body height was measured to the nearest 0.5 cm using a portable stadiometer (DT05L, Kinlee, Zhongshan Jinli Electronic Weighing Equipment Co. Ltd., China and Seca, Hamburg, Germany at the 1st and 2nd assessment, respectively), while body weight was measured to the nearest 0.1 kg using a digital scale (Inbody 720, Biospace Co., Seoul, Korea and Seca, Hamburg, Germany, respectively). Body weight was divided by the squared height to obtain the body mass index (BMI), expressed in kg/m^2^. We did not include body composition in these analyses as the devices were different at the two-time points, making a direct comparison difficult.

At both time points, isometric handgrip strength was recorded by assessing the maximum force (two trials of 3–4 s, the better recorded) on an adaptable dynamometer (JAMAR, Petterson Medical, Warrenville, IL, United States) using the self-reported dominant hand while seated (17). Physical performance was assessed by the 30-s chair-stand (for lower body), timed up-and-go (functional mobility) and 6-min walk (aerobic endurance) tests (18). The test–retest reliability of isometric handgrip strength and all physical performance assessments was found to be acceptable (ICC > 0.7) in the Kosovo population (19).

### 2.3 Genotyping and vitamin D status

The detailed methodology of the genotyping process was described in our previous study (12). Briefly, the saliva samples were collected and stored at −20°C in Kosovo, and then sent for further analyses to the Laboratory of Molecular Exercise Physiology at the Centre for Sport Science and University Sports, University of Vienna (Austria), where the samples were stored at −80°C. Genomic DNA was extracted from participants’ saliva samples using the GeneFiX™ Saliva-Prep DNA Isolation Kit (Isohelix, Kent, United Kingdom) following the manufacturer’s instructions. SNP genotyping was performed using commercially available genotyping assays for real-time quantitative PCR (Applied Biosystems/Thermo Fisher Scientific, Vienna, Austria). Specifically, for this study, genotyping of three candidate single nucleotide polymorphisms (SNPs) located in the VDR gene (rs7975232 [ApaI], rs2228570 [FokI] and rs731236 [TaqI]) and two in the GC gene (rs4588 and rs2282679) was performed.

Unfortunately, blood samples were only collected during the second assessment period. To minimize the impact of seasonal variations, measurements were carried out during the winter months, when the lowest values are expected due to the geographical location of Kosovo (42°40′N, 21°10′E). In order to minimize diurnal variations samples were taken in the morning and processed as described previously (14). Briefly, serum samples were first processed (centrifuged at 2,000 g for 10 min at room temperature) and stored (at −20°C) in Kosovo, and then shipped frozen to the laboratory in Vienna for measurement of total 25-(OH)D (including 25-OH vitamin D_2_ and 25-OH vitamin D_3_). Total serum 25-(OH)D was measured using a commercially available enzyme-linked immunosorbent assay kit (EUROIMMUN Medizinische Labordiagnostika AG, Lübeck, Germany). The assay demonstrated strong correlations with other methods: HPLC (*r*^2^ = 0.91, *n* = 80), LC–MS/MS (*r*^2^ = 0.93, *n* = 100), and the IDS 25-OH Vitamin D Direct EIA (*r*^2^ = 0.93, *n* = 231), consistent with the rigorous standards outlined by the Vitamin D Standardization Program. In this study, the intra-assay variability (CV) was 5.22% from 40 measurements per sample, and inter-assay variability (CV) was 7.82% from four measurements across 10 test runs.

### 2.4 Statistical analysis

All data analyses were performed using the SPSS 27 Windows statistical package (SPSS, Inc., Chicago, IL, United States). The significance level was set at *p* < 0.05. Descriptive data were presented as mean and standard deviation for continuous variables and relative frequencies for categorical variables. Two-way mixed ANOVA was used to determine the main effects of time (using the simple main effect for time) and group (using the simple main effect for groups), as well as time*group interactions. In the case of significant interactions, longitudinal changes were detected by dependent *t*-tests conducted separately for men and women. A non-parametric Kruskal–Wallis test followed by a Bonferroni-corrected *post hoc* test was used to determine differences in 25(OH)D concentrations between genotypes. Hardy–Weinberg equilibrium (HWE) was calculated by using a one-degree-of-freedom chi-squared test (*χ*^2^), comparing the observed distribution of genotypes with the distribution of genotypes expected from applying the Hardy–Weinberg equilibrium assumption [performed using the online application: http://www.ihh.kvl.dk/htm/kc/popgen/genetik/applets/kitest.htm (accessed on 2024-01-27)].

## 3 Results

### 3.1 Participants characteristics

A total of 308 participants were recruited in the first study, of whom 299 people provided saliva DNA samples (12). From this initial study population, 138 community-dwelling male and female participants aged ≥40 years from the Prishtina region agreed to participate in the second follow-up study. Age at baseline was 65.1 ± 9.0 years (63.7 ± 9.2 in female and 66.6 ± 8.7 in male participants). The time gap between the first and second assessments was 2.7 ± 0.3 years (2.9 ± 0.4 in female and 2.8 ± 0.3 in male participants). Figure 1 illustrates the participant flow and provides information on the reasons for attrition.

The overall and sex-specific characteristics of the study participants at baseline and follow-up are described in Table 1, whereby time, group (sex) and time*group interactions were reported. Male participants were significantly taller (*p* < 0.001) and had a greater body mass (*p* = 0.010), higher absolute handgrip and relative handgrip strength (*p* < 0.01), 6-min walk performance (*p* < 0.01) and nutritional status score (*p* < 0.05). Female participants were observed to have a significantly higher BMI (*p* < 0.01), a longer time to complete the timed up-and-go test (*p* < 0.010) and took a higher number of medications (*p* < 0.001).

**Table 1:** Longitudinal changes in anthropometric and physical fitness tests.

Variable	Overall baseline (*n* = 138)	Female baseline (*n* = 72)	Male baseline (*n* = 66)	Change to follow-up (overall) (*n* = 138)	Change to follow-up (female) (*n* = 72)	Change to follow-up (male) (*n* = 66)	Time	Sex	Time * sex
Height [m]	1.65 ± 0.09	1.59 ± 0.07	1.71 ± 0.07^###^	−0.010 ± 0.023°°°	−0.012 ± 0.026	−0.007 ± 0.019	**<0.001**	**<0.001**	0.246
Body mass [kg]	80.1 ± 14.0	77.1 ± 11.4	83.4 ± 15.9^##^	0.17 ± 4.25	0.16 ± 4.41	0.19 ± 4.10	0.631	**0.007**	0.975
BMI [kg/m^2^]	29.6 ± 4.9	30.7 ± 4.6	28.3 ± 4.9^##^	0.43 ± 1.70°°	0.55 ± 1.94	0.31 ± 1.38	**0.004**	**0.002**	0.397
Handgrip strength [kg]	31.7 ± 9.7	26.0 ± 6.1	38.0 ± 9.0^###^	−0.6 ± 5.2	−2.6 ± 4.6***	1.5 ± 4.9*	0.208	**<0.001**	**<0.001**
Handgrip strength relative [kg/kg]	0.40 ± 0.11	0.34 ± 0.09	0.46 ± 0.11^###^	−0.009 ± 0.070	−0.036 ± 0.062***	0.020 ± 0.067*	0.151	**<0.001**	**<0.001**
Timed up and go test [s]	6.66 ± 1.55	6.82 ± 1.56	6.49 ± 1.53^##^	0.95 ± 2.06°°°	1.43 ± 2.20***	0.42 ± 1.77	**<0.001**	**0.002**	**0.004**
30-s chair stand test [repetitions]	12 ± 3	12 ± 3	12 ± 3	−1 ± 3°°°	−1 ± 3	−1 ± 3	**<0.001**	0.158	0.729
6-min walk test [m]	451.0 ± 138.8	422.4 ± 129.4	481.8 ± 142.9^##^	−6.5 ± 88.7	−13.9 ± 89.1	1.4 ± 88.2	0.411	**0.001**	0.316
Mini nutritional status score [−]	24.8 ± 3.1	24.0 ± 3.4	25.8 ± 2.5^#^	1.2 ± 2.9°°°	2.1 ± 3.1***	0.3 ± 2.4	**<0.001**	**0.017**	**<0.001**
Number of medications [n]	1.8 ± 1.9	2.3 ± 1.9	1.3 ± 1.7^##^	0.55 ± 1.56°°°	0.45 ± 1.79	0.66 ± 1.27	**<0.001**	**0.003**	0.433

BMI (body mass index); Data are expressed as mean ± standard deviation for the total population and separately for men and women; two-way mixed ANOVA was used to determine main time and sex effects and time*sex interactions; ***p < 0. 001, *p < 0.05 for simple main effects (change over time separated by sex), °°°*p* < 0.001, °°*p* < 0.01 for main time effects, ###*p* < 0.001, ##*p* < 0.001, #*p* < 0.05 for main differences between males and females. Bold values indicate statistically significant p-values (*p* < 0.05).

Height decreased significantly over time (*p* < 0.001), whereas BMI (*p* = 0.004), nutritional status score, number of medications taken (p < 0.001) increased. A McNemar test showed that the prevalence of chronic diseases, particularly cardiovascular diseases (including hypertension), increased, whereas the prevalence of metabolic diseases like diabetes and osteoporosis stayed stable over the observation period. Regarding physical performance, absolute and relative handgrip strength (*p* < 0.001), timed up and go test (*p* = 0.002) and 6-min walk test (*p* = 0.001) worsened, whereas the 30-s chair stand test remained unchanged (*p* = 0.158). A time*sex interaction was observed for handgrip strength and timed up and go test. Follow-up analyses showed that a significant decrease in handgrip strength and handgrip strength relative to individual body mass was observed in women (*p* < 0.001), but the opposite occurred in men (*p* < 0.05). The time taken to complete the timed up-and-go test increased significantly over time only in female participants (*p* < 0.001), whereas the increase in men was smaller and did not reach significance. Interestingly, the mini nutritional status score increased only in women (*p* < 0.001). The increase in BMI observed in both women and men did not correlate with the changes in any of the fitness parameters (*p* > 0.05).

### 3.2 Genotyping and vitamin D levels (at time point 2)

The genotype distribution for Apal (rs7975232), Fokl (rs2228570), Taql (rs731236), rs4588 and rs2282679 and the Hardy–Weinberg equilibrium (HWE) of these genotypes are shown in Table 2. All SNPs investigated were in Hardy–Weinberg equilibrium (HWE), except for Fokl (rs2228570).

**Table 2:** Genotype frequencies.

SNP	Aliases	Chr	Gene	Major/Minor alleles	Subjects (n)	Genotype frequency, *n* (%)	Allele frequency, *n* (%)	HWE
rs7975232	ApaI	12	VDR	A/C	128	AA 42 (32.8) AC 67 (52.3) CC 19 (14.9)	A 74.5 (58.98) C 52.5 (41.02)	0.652
rs2228570	FokI	12	VDR	G/A	131	AA 19 (14.5) AG 44 (33.6) GG 68 (51.9)	A 41 (31.30) G 90 (68.70)	0.043*
rs731236	TaqI	12	VDR	A/G	129	AA 51 (39.5) AG 55 (42.6) GG 23 (17.8)	A 78.5 (60.85) G 50.5 (39.15)	0.490
rs4588	–	4	GC	C/A	130	CC 82 (63.1) CA 40 (30.7) AA 8 (6.2)	C 102 (78.5) A 28 (21.5)	0.593
rs2282679	–	4	GC	A/C	131	CC 9 (6.9) CA 40 (30.5) AA 82 (62.6)	C 29 (22.14) A 102 (77.86)	0.425

SNP (single nucleotide polymorphism), Chr (chromosome), HWE (Hardy–Weinberg Equilibrium); *Distribution not consistent with Hardy Weinberg’s law at *p* < 0.05.

**Figure 2:**
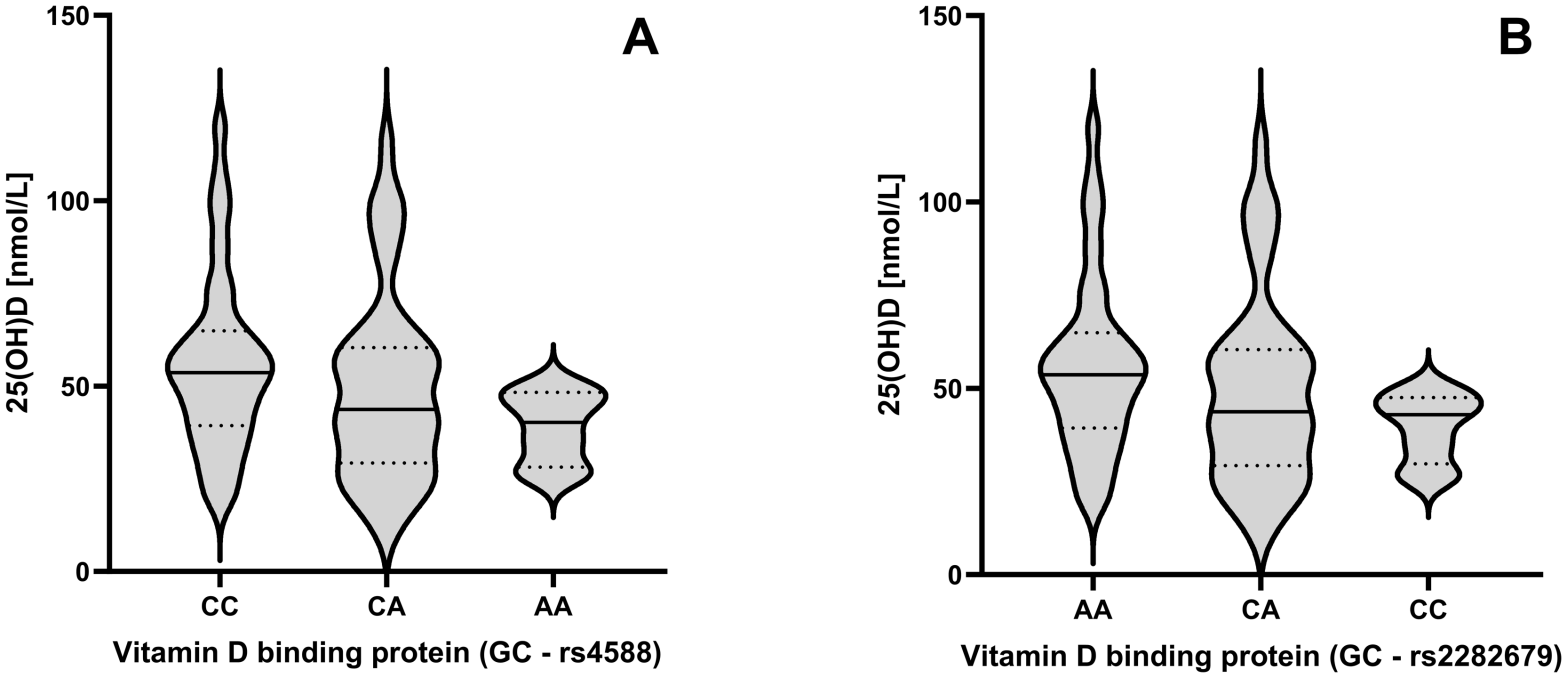
Impact of the rs4588 **(A)** and rs2282679 **(B)** genotypes across GC genes on 25-hydroxyvitamin D (25(OH)D) levels. Violine plots are shown to visualize the distribution of the data with 25th, 50th and 75th percentile shown as lines.

A Kruskal-Wallis test revealed significantly different 25(OH)D levels between genotypes of the GC gene for rs4588 [*χ*^2^(2) = 6.509, *p* = 0.039] and rs2282679 [χ^2^(2) = 6.648, *p* = 0.036]. For rs4588, median 25(OH)D levels were lower in individuals with the AA genotype (40.2 [26.2–45.5] nmol/L) compared with those with the CC genotype (53.6 [17.7–120.6] nmol/L), although a Bonferroni-corrected *post hoc* analysis did not reach significance between the two groups (*p* = 0.076). In contrast, for rs2282679, median 25(OH)D levels were lower in individuals with the CC genotype (42.9 [26.2–49.5] nmol/L) compared with those with the AA genotype (53.6 [17.7–120.6] nmol/L). Again, a Bonferroni-corrected *post hoc* test did not reach significance (*p* = 0.076). Differences in 25(OH)D levels between GC genotypes are shown in Figure 2. No differences were found for the genotypes in the VDR gene, with ApaI [*χ*^2^(2) = 1.769, *p* = 0.413], FokI [*χ*^2^(2) = 4.897, *p* = 0.086] and TaqI [*χ*^2^(2) = 0.393, *p* = 0.822].

### 3.3 Impact of VDR genotypes on longitudinal changes in physical performance

Baseline data and longitudinal changes in physical performance health-related parameters are shown in Table 3 separately for ApaI (rs7975232), FokI (rs2228570), and TaqI (rs731236).

**Table 3:** Influence of vitamin D receptor (VDR) genetic variants on changes in physical performance and health-related parameters up to follow-up.

Variable	Baseline			Change to follow-up			*p*-values		
rs7975232 [Apal]	AA (*n* = 42)	AC (*n* = 67)	CC (*n* = 19)	AA (*n* = 42)	AC (*n* = 67)	CC (*n* = 19)	Time	Group	Time * Apal
BMI [kg/m^2^]	31.4 ± 5.9^a^	28.9 ± 4.3^a,b^	28.5 ± 3.7^b^	−0.05 ± 1.65	0.50 ± 1.65°	1.23 ± 1.92°	**0.001**	**0.043**	**0.023**
Handgrip strength [kg]	29.5 ± 11.6	32.1 ± 8.4	30.3 ± 5.9	−1.26 ± 5.5	0.47 ± 4.93	−3.98 ± 4.06°°°	**0.002**	0.106	**0.003**
Handgrip strength relative [kg/kg]	0.36 ± 0.14^a^	0.41 ± 0.10^b^	0.40 ± 0.09^a,b^	−0.01 ± 0.07	0.00 ± 0.07	−0.06 ± 0.06°°°	**<0.001**	**0.011**	**<0.001**
Timed up and go test [s]	6.78 ± 1.77	6.60 ± 1.52	6.58 ± 1.49	1.47 ± 2.65°°	0.73 ± 1.86°°	0.96 ± 1.46°	**<0.001**	0.219	0.200
30-s chair stand test [repetitions]	11 ± 3^a^	12 ± 2^a,b^	13 ± 3^b^	−1 ± 3°°	−1 ± 3	−1 ± 3	**0.005**	**0.008**	0.641
6-min walk test [m]	425.4 ± 153.2	453.6 ± 132.0	469.5 ± 116.5	−8.8 ± 98.8	0.4 ± 86.0	−25.9 ± 90.2	0.217	0.343	0.530
Mini nutritional status score [−]	24.6 ± 3.4	24.8 ± 3.0	25.4 ± 2.5	0.85 ± 3.24	1.48 ± 2.76°°°	1.05 ± 2.36	**<0.001**	0.234	0.520
Number of medications [n]	2.1 ± 1.8	1.8 ± 1.8	1.7 ± 1.9	0.42 ± 1.70	0.63 ± 1.58°°	0.61 ± 1.42	**0.001**	0.764	0.790

Variable	Baseline			Change to follow-up			*p*-values		
rs2228570 [Fokl]	AA (*n* = 19)	AG (*n* = 44)	GG (*n* = 68)	AA (*n* = 19)	AG (*n* = 44)	GG (*n* = 68)	Time	Group	Time * Fokl
BMI [kg/m^2^]	30.3 ± 4.7	29.8 ± 5.2	29.2 ± 4.9	0.04 ± 1.82	0.38 ± 1.56	0.60 ± 1.81	0.052	0.786	0.430
Hand grip strength [kg]	30.0 ± 10.4	32.9 ± 10.0	30.5 ± 8.6	−0.81 ± 5.56	0.27 ± 4.76	−1.08 ± 5.51	0.310	0.222	0.412
Hand grip strength relative [kg/kg]	0.37 ± 0.12	0.41 ± 0.12	0.39 ± 0.11	−0.00 ± 0.07	0.00 ± 0.06	−0.02 ± 0.07	0.308	0.363	0.303
Timed up and go test [s]	6.13 ± 1.78	6.86 ± 1.64	6.69 ± 1.46	1.41 ± 1.90°°	0.48 ± 1.89	1.20 ± 2.25°°°	**<0.001**	0.552	0.136
30-s chair stand test [repetitions]	12 ± 2	12 ± 3	11 ± 3	−0 ± 3	−1 ± 3	−1 ± 3°	**0.024**	0.164	0.647
6-min walk test [m]	444.0 ± 103.8	481.8 ± 156.8	429.0 ± 129.0	6.9 ± 75.1	−15.8 ± 84.8	−4.0 ± 97.8	0.637	0.133	0.631
Mini nutritional status score [−]	25.2 ± 2.4	25.0 ± 3.4	24.5 ± 3.1	0.97 ± 2.42	1.48 ± 3.04°°	1.15 ± 3.02°°	**<0.001**	0.288	0.776
Number of medications [n]	1.8 ± 1.7	1.4 ± 1.7	1.9 ± 1.8	0.79 ± 1.75	0.52 ± 1.41°	0.55 ± 1.67°	**<0.001**	0.217	0.814

Variable	Baseline			Change to follow-up			*p*-values		
rs731236 [Taql]	AA (*n* = 51)	AG (*n* = 55)	GG (*n* = 23)	AA (*n* = 51)	AG (*n* = 55)	GG (*n* = 23)	Time	Group	Time * Taql
BMI [kg/m^2^]	28.6 ± 3.9	29.9 ± 5.2	31.6 ± 5.6	0.82 ± 1.80°°	0.44 ± 1.48°	−0.23 ± 2.05	**0.038**	0.096	0.055
Hand grip strength [kg]	30.2 ± 8.0	32.5 ± 9.6	29.3 ± 11.3	−1.33 ± 5.21	−0.02 ± 5.54	−0.95 ± 4.62	0.127	0.180	0.429
Hand grip strength relative [kg/kg2]	0.39 ± 0.10	0.40 ± 0.11	0.36 ± 0.14	−0.28 ± 0.07	0.00 ± 0.07	−0.01 ± 0.06	0.109	0.232	0.099
Timed up and go test [s]	6.59 ± 1.42	6.71 ± 1.61	6.74 ± 1.92	0.78 ± 1.55°°	0.87 ± 1.89°°	1.92 ± 3.26°	**<0.001**	0.209	0.074
30-s chair stand test [repetitions]	12 ± 3	11 ± 3	11 ± 3	−1 ± 3°	−0 ± 3	−2 ± 2°°	**0.001**	0.099	0.196
6-min walk test [m]	450.2 ± 131.9	449.4 ± 138.1	422.6 ± 142.9	2.8 ± 89.0	−20.6 ± 92.8	8.5 ± 85.2	0.721	0.702	0.290
Mini nutritional status score [−]	25.1 ± 3.5	24.5 ± 2.6	24.8 ± 3.7	1.16 ± 3.01°°	1.56 ± 2.75°°°	0.65 ± 3.34	**<0.001**	0.514	0.460
Number of medications [n]	1.6 ± 1.8	1.8 ± 1.8	2.3 ± 2.0	0.72 ± 1.39°°	0.49 ± 1.60°	0.36 ± 2.04	**0.001**	0.461	0.629

BMI (body mass index). Data are expressed as means ± standard deviations for the participants separated based on the genotypes; Mixed two-way ANOVA was used to determine main time and group effects as well as time*group interactions; °°°*p* < 0.001, °°*p* < 0.01 for main time effects, different superscript letters (“a” and “b”) indicate the statistically significant differences for simple main effects between groups. Bold values indicate statistically significant p-values (*p* < 0.05).

For ApaI, there was a statistically significant interaction between ApaI and time on BMI [*F*(2,125) = 3.870, *p* = 0.023, partial *η*^2^ = 0.058], handgrip strength [*F*(2,125) = 6.141, *p* = 0.003, partial *η*^2^ = 0.089] and handgrip strength related to body mass [*F*(2,125) = 7,528, *p* < 0.001, partial *η*^2^ = 0.108]. BMI increased, but absolute and relative handgrip strength decreased in CC carriers, whereas these parameters remained unchanged in the AA genotype group. The interaction between ApaI genotype and change in handgrip strength remained significant when controlled for age, change in BMI, and 25(OH)D level [*F*(2,121) = 7.733, *p* < 0.001, partial *η*^2^ = 0.113].

There was no statistically significant interaction for the 30-s chair stand test [*F*(2,124) = 0.447, *p* = 0.641, partial *η*^2^ = 0.007], but the main effect of group showed that there was an overall significant difference in mean 30-s chair stand repetitions between genotype groups [*F*(2,124) = 5.006, *p* = 0.008, partial *η*^2^ = 0.075]. Interestingly, CC carriers had significantly higher values than AA carriers (*p* = 0.035). Results for the 6-min walk test showed no effect of the ApaI genotype, either at baseline or with respect to changes over time.

For FokI and TaqI, no genotype*time interactions or main group effects were detected (Table 3).

### 3.4 Impact of GC genotypes on longitudinal changes in physical performance

Similar to FokI and TaqI, no genotype*time interactions or main group effects were found for rs4588 and rs2282679 in the GC gene (Table 4).

**Table 4:** Influence of vitamin D binding protein (GC) genetic variants on changes to follow-up.

Variable	Baseline			Change to follow-up			*P*-values		
rs4588	CC (*n* = 82)	CA (*n* = 40)	AA (*n* = 8)	CC (*n* = 82)	CA (*n* = 40)	AA (*n* = 8)	Time	Group	Time * GC1
BMI [kg/m^2^]	29.6 ± 4.9	29.8 ± 5.1	30.3 ± 4.6	0.34 ± 1.83	0.68 ± 1.56°°	0.60 ± 1.75	**0.023**	0.833	0.592
Handgrip strength [kg]	31.3 ± 9.5	30.7 ± 9.7	31.1 ± 6.1	−1.33 ± 5.02	0.66 ± 5.75	−1.20 ± 3.70	0.374	0.978	0.137
Handgrip strength relative [kg/kg]	0.39 ± 0.11	0.39 ± 0.12	0.39 ± 0.08	−0.02 ± 0.07	0.00 ± 0.08	−0.02 ± 0.05	0.193	0.921	0.288
Timed up and go test [s]	6.63 ± 1.52	6.82 ± 1.68	6.19 ± 1.79	1.06 ± 2.31°°°	0.92 ± 1.72°°	0.80 ± 1.84	**0.001**	0.551	0.905
30-s chair stand test [repetitions]	12 ± 3	11 ± 3	12 ± 2	−1 ± 3°°	−0 ± 3	−2 ± 3	**0.004**	0.307	0.325
6-min walk test [m]	454.3 ± 128.4	425.3 ± 161.5	476.8 ± 79.4	−8.7 ± 93.2	−4.6 ± 87.2	2.3 ± 79.4	0.762	0.366	0.933
Mini nutritional status score [−]	24.7 ± 3.2	24.9 ± 3.3	24.1 ± 2.6	1.24 ± 2.92°°°	1.15 ± 2.99°	1.75 ± 3.40	**0.001**	0.822	0.873
Number of medications [n]	1.7 ± 1.8	2.1 ± 1.9	1.75 ± 1.39	0.71 ± 1.63°°°	0.18 ± 1.48	1.13 ± 1.55	**0.002**	0.795	0.131

Variable	Baseline			Change to follow-up			*P*-values		
rs2282679	CC (*n* = 9)	CA (*n* = 40)	AA (*n* = 82)	CC (*n* = 9)	CA (*n* = 40)	AA (*n* = 82)	Time	Group	Time * GC2
BMI [kg/m^2^]	30.8 ± 4.5	29.8 ± 5.1	29.6 ± 4.9	0.50 ± 1.67	0.68 ± 1.56°°	0.34 ± 1.83	**0.026**	0.705	0.604
Hand grip strength [kg]	34.6 ± 11.8	30.7 ± 9.7	31.3 ± 9.5	−1.29 ± 3.47	0.66 ± 5.75	−1.33 ± 5.02	0.329	0.658	0.133
Hand grip strength relative [kg/kg]	0.41 ± 0.09	0.39 ± 0.12	0.39 ± 0.11	−0.02 ± 0.04	0.00 ± 0.08	−0.02 ± 0.07	0.170	0.906	0.281
Timed up and go test [s]	6.42 ± 1.82	6.82 ± 1.68	6.63 ± 1.52	0.72 ± 1.73	0.92 ± 1.72°°	1.06 ± 2.31°°°	**0.001**	0.712	0.867
30-s chair stand test [repetitions]	11 ± 3	11 ± 3	12 ± 3	−2 ± 3	0 ± 3	−1 ± 3°°	**0.007**	0.208	0.432
6-min walk test [m]	454.9 ± 99.1	425.3 ± 161.5	454.3 ± 128.4	2.6 ± 74.2	−4.6 ± 87.2	−8.7 ± 93.2	0.758	0.488	0.924
Mini nutritional status score [−]	24.6 ± 2.7	24.9 ± 3.3	24.7 ± 3.2	1.67 ± 3.19	1.15 ± 2.99°	1.24 ± 2.92°°°	**0.001**	0.931	0.894
Number of medications [n]	1.7 ± 1.3	2.1 ± 1.9	1.7 ± 1.8	1.11 ± 1.45	0.18 ± 1.48	0.71 ± 1.63°°°	**0.001**	0.832	0.125

BMI (body mass index). Data are expressed as means ± standard deviations for the participants separated based on the genotypes; Mixed two-way ANOVA was used to determine main time and group effects as well as time*group interactions; °°°*p* < 0.001, °°*p* < 0.01 for simple main time effects. Bold values indicate statistically significant p-values (*p* < 0.05).

In detail, there was no interaction for the rs4588 genotypes and time with respect to BMI [*F*(2, 127) = 0.526, *p* = 0.592, partial *η*^2^ = 0.008], handgrip strength [*F*(2,127) = 2.017, *p* = 0.137, partial *η*^2^ = 0.031], timed up and go [*F*(2,127) = 0.100, *p* = 0.905, partial *η*^2^ = 0.002], 30-s chair stand test [*F*(2,126) = 1.134, *p* = 0.325, partial *η*^2^ = 0.018] and 6-min walk test [*F*(2,126) = 0.070, *p* = 0.933, partial *η*^2^ = 0.001]. However, simple main effects for time revealed that only CC and CA carriers were significantly slower during the timed up and go test (*p* < 0.05) hinting to an unfavorable AA genotype.

For the rs2282679, there was no interaction effect with time for BMI [*F*(2,128) = 0.505, *p* = 0.604, partial *η*^2^ = 0.008], handgrip strength [*F*(2,128) = 2.052, *p* = 0.133, partial *η*^2^ = 0.031], timed up and go [*F*(2,128) = 0.143, *p* = 0.867, partial *η*^2^ = 0.002], 30-s chair stand test [*F*(2,127) = 0.846, *p* = 0.432, partial *η*^2^ = 0.013] and 6-min walk test [*F*(2,127) = 0.079, *p* = 0.924, partial *η*^2^ = 0.001]. Similar to the findings above, simple main effects for time showed that time needed for the timed up and go test only increased in CA and AA carriers (*p* < 0.05) hinting to an unfavorable CC genotype.

## 4 Discussion

This study shows that muscle strength and physical performance decline over approximately 3 years, regardless of biological sex, although isometric strength and functional mobility decline at a higher rate in female participants. In addition, vitamin D-related gene polymorphisms may play a role in this decline, with rs7975232 (Apal) emerging as a promising candidate regarding its influence on strength-related traits. Beyond this observation, 25(OH)D concentration at time point 2 was significantly lower in AA carriers of the rs4588 and in CC carriers of the rs2282679 SNPs, both located in the vitamin D binding GC gene.

It has been suggested that the age-related decline in muscle strength should not necessarily be viewed as a singular process but rather as a more comprehensive multifactorial one (e.g., inability of the nervous systems to fully activate skeletal muscles, differences between biological sexes, etc.). Furthermore, a significant association between low levels of muscle strength and poor physical performance and/or physical disability has been reported (20), with potential implications for morbidity and mortality. The results of this study confirm the age-related decline in isometric muscle strength relative to the individual’s body mass, but only in female participants. It is noteworthy that a very wide age range was included in this study (40 years and older) with 8.7% of the participants under 50 years of age, 60.1% of the participants between 50 and 70 years of age, and 31.2% of the participants being over 70 years of age. The distribution of women and men between the age groups is similar and not statistically different. Therefore, the observed difference between men and women could be explained by an earlier onset of handgrip strength decline in women, as shown in the Copenhagen Sarcopenia Study, where handgrip strength declined in women from the age of 50 and in men from the age of 60 years (21). It has been suggested that poorer physical function in women compared with men can be explained predominantly by differences in body composition. The higher proportion of body fat in women may put them at a significant biomechanical disadvantage resulting in greater disability in old age (22). As BMI is significantly higher in our female cohort this could be a further factor for the observed differences.

However, the primary aim of this study was to investigate the potential influence of SNPs from vitamin D pathway-related candidate genes (VDR and GC) on decline in physical performance and strength. The selection of the genotypes was based on a previous systematic review showing some evidence for their association with vitamin D levels and some extent also to muscular performance (5). The VDR gene encodes the vitamin D receptor, which is a member of the nuclear receptor superfamily and is a genomic and non-genomic mediator of the effects of vitamin D in the organism, whereas the GC gene encodes the vitamin D binding protein, which is a multifunctional protein that also has a role as a transporter of vitamin D metabolites (23).

In the VDR gene, rs7975232 (Apal), an intron variant, emerged as a promising candidate in this regard, with time and group effects and time*group interactions observed for grip strength. A significant decline was seen in homozygous recessive (CC) genotype carriers for handgrip strength and relative handgrip strength, and in homozygous dominant (AA) genotype carriers for the 30-s chair stand test. Consistent with these findings, cross-sectional studies have reported that carriers of the homozygous dominant genotype have a significantly higher handgrip strength (24), but also that homozygous recessive carriers have significantly higher knee and elbow peak torque (25). As observed, both upper and lower body strength were inversely affected by ApaI genotypes, suggesting a beneficial role of the major allele in upper strength and the minor allele in lower body strength. However, these findings warrant further mechanistic investigation. Among carriers of rs2228570 [FokI] genotypes (initiator codon variant), subjects homozygous for the major allele (GG) showed a trend for a decreased chair stand performance, suggesting that the minor allele could be the beneficial one for lower body strength. This would be in line with previous studies observing that carriers of the major homozygous genotype had significantly lower quadriceps strength (26) or significantly lower peak and average isometric quadriceps strength (27). However, other studies found no significant associations between these genotypes and muscle properties (28, 29). Finally, the rs731236 [TaqI] polymorphism, a synonymous variant, showed no effect on changes in physical performance in our study. Studies in this area also remain controversial, as studies showing no association between TaqI genotypes and muscular performance (25, 28) contrast with others observing higher isometric quadriceps strength in homozygous recessive genotypes compared with homozygous dominant and heterozygous genotypes (27) or higher handgrip strength in centenarians with dominant genotypes compared with the other genotypes (24).

For the GC-investigated SNPs, our study shows that the decline in TUG performance and chair-stand test was pronounced in carriers of homozygous dominant genotypes in both rs4588 and rs2282679 polymorphisms. This suggests that the beneficiary allele in this population is the minor allele, T allele for rs4588 and G allele for rs2282679, respectively. To the best of our knowledge, the literature investigating the direct impact of these polymorphisms across GC gene in muscle traits is sparse, and our results seem promising although these findings need to be validated in larger study populations, especially as carriers of the rare genotype comprised only 8 (rs4588) and 9 (rs2282679) participants.

In our previous systematic review, we demonstrated that certain genetic variants within genes involved in the vitamin D pathway influence vitamin D levels (5). Vitamin D deficiency is considered to be a widespread global health problem, particularly in older populations (30). A potential association between vitamin D levels and muscle strength and/or performance is of great interest not only to the scientific community but also to public health professionals, although direct positive effects of vitamin D supplementation on sarcopenia indices remain controversial (31). Individual genetic makeup may add to this equation. In this study, carriers of the major alleles for rs4588 and rs2282679 (located within the GC gene) were observed to have slightly higher serum 25(OH)D levels, although the significance did not persist after Bonferroni-correction of *post hoc* analyses. It is unlikely that BMI, weight, or age have driven this observation, as these parameters were not different between the genotype groups. Furthermore, these results are consistent with many of those included in our systematic review (5), where 73 and 77% of the conducted studies selected (respectively) showed an association of rs4588 and rs2282679 with vitamin D levels, and the major alleles were the beneficiary ones in both cases. This was not evident between selected SNPs across the VDR gene, with conflicting results already reported (5). However, other factors should be taken into consideration when comparing such data between the different studies, including the heterogeneity in measurements of vitamin D status or levels and participant characteristics.

The decline in age-related muscle strength is recognized as a multifactorial process influenced by genetics, but also lifestyle factors (such as nutritional and physical behaviors) as well as metabolic processes that may have impacted the study outcomes. Regarding vitamin D supplementation, data collected and reported for the studied population (14), revealed that 87.6% of participants did not take vitamin D supplements, despite their very low daily dietary vitamin D intake (1.89 ± 0.67 μg/day). It further appears from the evidence from systematic reviews and meta-analyses that physical activity alone may not be sufficient to increase 25(OH)D levels, regardless of the amount of time spent outdoors (32). As no specific data on physical activity levels were recorded, it was assumed that participants did not significantly alter their physical activity behaviors over time. To address potential confounding factors, the Physical Readiness Questionnaire (PAR-Q) was used to exclude severe cases, along with the original study’s specific exclusion criteria (14), which included acute illness preventing exercise testing and serious chronic illnesses requiring continuous medical care. Additionally, a comparison of the two measurement time points showed no changes in the prevalence of self-reported medical conditions such as osteoporosis, hypertension, or diabetes.

To further investigate these effects, we performed correlations between BMI and 25(OH)D levels, as well as between longitudinal changes in BMI and muscle strength. However, no significant associations were found. This lack of association may reflect the complex interplay of factors influencing vitamin D status, including the known role of adiposity in the sequestration of vitamin D, which can reduce its bioavailability. While body fat is an important factor in aging, and not BMI alone, our study did not find any significant correlation between BMI, weight, or body composition with vitamin D levels. Despite the recognized influence of adiposity on vitamin D metabolism (33, 34), our findings suggest that other factors may be at play in this cohort. In addition, in contrast to height, which declined significantly from the first to the second measurement, weight did not change, thus resulting in an overall increase in BMI over time. This reflected in the increased prevalence of overweight and obesity in the study population over time (from 40.6 to 42.0% in the overweight group and from 42.0 to 44.9% in the obese group), particularly in women (from 38.9 to 40.3% and from 50.0 to 55.6%, respectively). The number of participants with normal weight decreased from 16.7 to 12.3% compared to the baseline measurements, which raises the alarm about the seriousness of this problem for the public health of Kosovo (11, 12). It should be noted that the optimal BMI levels may be higher for older adults as compared to young adults (35), and that body composition may be a better indicator of health (36). Future studies need to address this issue in the region in more detail. A recent review of the global burden of metabolic diseases for the period 2000–2019 found that the prevalence of metabolic diseases (including obesity) is increasing worldwide, regardless of socio-demographic index (37). This, along with the low expected life expectancy observed in Kosovo (70.3 years for men and 74.8 years for women) (38) may reflect the impact of increasing overweight and obesity rates.

Given that the expected life expectancy and the proportion of older people are higher in high-income countries, most age-related studies are conducted in these parts of the world. Nevertheless, the expected growth of the older population from developing countries is estimated to outstrip that from high-income countries by a ratio of 2 to 1 by 2050 (39), raising many concerns about what the future holds. This is particularly worrying given the lack of data on aging and age-related decline in muscle phenotypes in low-and-middle income countries, and the prevalence of age-related diseases that are expected to increase as an inevitable burden of the future (40).

Although this study was designed, planned, and conducted to the best of our knowledge, there are certain limitations. Firstly, the number of participants included in the study is rather low. Of the 308 subjects initially recruited for the genotype analyses (12), only 138 were able or wanted to participate in the follow up study. This limitation could have been influenced by the generally lower number of mature adults (8.1%), a lower life expectancy at birth (41) and the low socio-economic level of this age group in Kosovo (42). Secondly, the focus was on middle-aged to older adults, given the significant and visible decline in physical performance that is observed in these age groups. While this demographic provided a valuable opportunity to study the early and more pronounced stages of muscle strength decline, which aligns with our core research aims, it could be considered a limitation. Future long-term studies should examine the effects of genetic variants in vitamin D pathway-related genes on muscle characteristics from young adulthood into later life.

In conclusion, this study provides valuable insights into the age-related decline in muscle strength and physical performance in a developing European country. On of the key strengths of this research is its focus on a diverse cohort, including a broad age range of middle-aged and older adults, which enabled the examination of the early stages of muscle strength decline. Furthermore, the inclusion of participants from a lower socio-economic background and a developing country context provides unique insights and emphasizes the necessity for region-specific interventions. Additionally, the implication and interaction of genotypes across vitamin D-related genes, specifically rs7975232 (Apal) in the VDR gene and rs4588 and rs2282679 in the GC gene, along with vitamin D status (alone and in combination) should help prevent future complications by paving the way for tailored-based individual and population-specific intervention strategies. These findings represent one of the few follow-up data sets on the age-related effects of muscle phenotypes and the genetic background within the population of Kosovo, highlighting the importance of incorporating genetic, socio-economic, and regional factors into public health strategies aimed at improving the well-being of aging populations.

## Data Availability

The datasets presented in this study can be found in online repositories. The names of the repository/repositories and accession number(s) can be found: https://data.mendeley.com/datasets/jcfjfh8ybk/1, doi: 10.17632/jcfjfh8ybk.1.
